# Supplementary material for: HIVprotI: an integrated web based platform for prediction and design of HIV proteins inhibitors
Source: J Cheminform. 2018 Mar 9;10:12. doi: 10.1186/s13321-018-0266-y (PMC5845081; doi:10.1186/s13321-018-0266-y)
Supplement: Supplementary file 2 — Additional file 2. Source code of HIVProtI web server. [file 13321_2018_266_MOESM2_ESM.zip › HIVprotI_Source-code/hivprotI/weka-3-6-6/doc/weka/gui/experiment/package-summary.html]

weka.gui.experiment


---


|  |  |  |
| --- | --- | --- |
| **Overview** \| **Package** \| Class \| **Tree** \| **Deprecated** \| **Index** \| **Help** \| **Weka's home** | |  |
| **PREV PACKAGE**   **NEXT PACKAGE** | **FRAMES**    **NO FRAMES**     **All Classes** |  |


---

## Package weka.gui.experiment

| **Class Summary** | |
| --- | --- |
| **AlgorithmListPanel** | This panel controls setting a list of algorithms for an experiment to iterate over. |
| **DatasetListPanel** | This panel controls setting a list of datasets for an experiment to iterate over. |
| **DistributeExperimentPanel** | This panel enables an experiment to be distributed to multiple hosts; it also allows remote host names to be specified. |
| **Experimenter** | The main class for the experiment environment. |
| **ExperimenterDefaults** | This class offers get methods for the default Experimenter settings in the props file `weka/gui/experiment/Experimenter.props`. |
| **GeneratorPropertyIteratorPanel** | This panel controls setting a list of values for an arbitrary resultgenerator property for an experiment to iterate over. |
| **HostListPanel** | This panel controls setting a list of hosts for a RemoteExperiment to use. |
| **Messages** | Messages. |
| **OutputFormatDialog** | A dialog for setting various output format parameters. |
| **ResultsPanel** | This panel controls simple analysis of experimental results. |
| **RunNumberPanel** | This panel controls configuration of lower and upper run numbers in an experiment. |
| **RunPanel** | This panel controls the running of an experiment. |
| **SetupModePanel** | This panel switches between simple and advanced experiment setup panels. |
| **SetupPanel** | This panel controls the configuration of an experiment. |
| **SimpleSetupPanel** | This panel controls the configuration of an experiment. |

---


|  |  |  |
| --- | --- | --- |
| **Overview** \| **Package** \| Class \| **Tree** \| **Deprecated** \| **Index** \| **Help** \| **Weka's home** | |  |
| **PREV PACKAGE**   **NEXT PACKAGE** | **FRAMES**    **NO FRAMES**     **All Classes** |  |


---
